# Supplementary material for: Mechanisms governing the pioneering and redistribution capabilities of the non-classical pioneer PU.1
Source: Nat Commun. 2020 Jan 21;11:402. doi: 10.1038/s41467-019-13960-2 (PMC6972792; doi:10.1038/s41467-019-13960-2)
Supplement: Supplementary file 7 — Source data [file 41467_2019_13960_MOESM7_ESM.zip › Source_Data/Figure6/Figure6B_MotifScanOutput/ETS1_PU.1/homerResults/motif8.similar.html]

motif8

## Information for motif8

G
T
A
C
T
C
A
G
A
T
C
G
A
G
T
C
A
G
T
C
C
T
G
A
G
T
A
C
C
G
T
A
  
Reverse Opposite:  

C
G
A
T
C
A
T
G
A
G
C
T
A
C
T
G
A
C
T
G
A
T
G
C
A
G
T
C
C
A
T
G
  

|  |  |
| --- | --- |
| p-value: | 1e-216 |
| log p-value: | -4.995e+02 |
| Information Content per bp: | 1.774 |
| Number of Target Sequences with motif | 4436.0 |
| Percentage of Target Sequences with motif | 28.02% |
| Number of Background Sequences with motif | 5008.1 |
| Percentage of Background Sequences with motif | 15.62% |
| Average Position of motif in Targets | 100.7 +/- 53.0bp |
| Average Position of motif in Background | 100.3 +/- 63.1bp |
| Strand Bias (log2 ratio + to - strand density) | -0.1 |
| Multiplicity (# of sites on avg that occur together) | 1.11 |
| Motif File: | file (matrix) reverse opposite |

### Similar de novo motifs found

|  |  |  |  |  |  |  |  |
| --- | --- | --- | --- | --- | --- | --- | --- |
| Rank | Match Score | Redundant Motif | P-value | log P-value | % of Targets | % of Background | Motif file |
| 1 | 0.891 | G A T C G T A C A C T G G T A C A G T C A G T C G T C A G A T C C G T A | 1e-167 | -386.397604 | 25.12% | 14.59% | motif file (matrix) |
| 2 | 0.771 | G T A C G A T C C A G T G T A C A G T C A G T C G T A C A G T C C T G A A G T C | 1e-147 | -340.559813 | 21.42% | 12.17% | motif file (matrix) |
| 3 | 0.930 | A C T G A T G C A G T C A G T C C G T A A G T C C G T A | 1e-76 | -175.493771 | 21.00% | 14.22% | motif file (matrix) |
